# Error Analysis of Narrowband Power-Line Communication in the Off-Grid Electrical System

**DOI:** 10.3390/s22062265

**Published:** 2022-03-15

**Authors:** Vojtech Blazek, Zdenek Slanina, Michal Petruzela, Roman Hrbáč, Jan Vysocký, Lukas Prokop, Stanislav Misak, Wojciech Walendziuk

**Affiliations:** 1 ENET Centre, VSB—Technical University of Ostrava, 17. Listopadu 2172/15, Poruba, 708 00 Ostrava, Czech Republic; vojtech.blazek@vsb.cz (V.B.); petruzelam@gmail.com (M.P.); jan.vysocky@vsb.cz (J.V.); lukas.prokop@vsb.cz (L.P.); stanislav.misak@vsb.cz (S.M.); 2 Faculty of Electric Engineering and Computer Science, VSB—Technical University of Ostrava, 17. Listopadu 2172/15, Poruba, 708 00 Ostrava, Czech Republic; zdenek.slanina@vsb.cz (Z.S.); roman.hrbac@vsb.cz (R.H.); 3 Faculty of Electrical Engineering, Bialystok University of Technology, Wiejska 45D, 15-351 Bialystok, Poland

**Keywords:** power line communication, off-grid, microgrid

## Abstract

Narrowband power-line communication seems to be a suitable communication technology designed for off-grid renewable energy solutions. Existing electrical installations can be designed both for the transmission of electricity and for the communication of electrical equipment operating inside such an installation. This study presents an implementation of the above-mentioned off-grid communication system and examines the basic problems related to its exploitation. The authors of this article focused their attention primarily on examining the disturbance of the communication channel caused by the use of typical electrical devices, such as: a light bulb, a kettle, etc. used in a household. The aim of the research was also to find the impact of switching on individual devices and their combinations on the disturbances during data transmission. Measurements of incorrectly transmitted data packets were carried out and then the test results were referred to the error measures. Moreover, the influence of the carrier frequencies on the signal attenuation and the method of eliminating the existing interferences were also discussed.

## 1. Introduction

Although power-line communication (PLC) is a relatively old technology and has been widely applied in some technical fields for decades, a real boom in research of PLC technology came in the 1990s. This increase in research activities was mainly connected with the applicability of PLC in smart grid (SG) electrical systems [1,2,3,4,5].

The first ideas of transferring data over power lines [6] were created during the first half of the 19th century, and the first realization of this idea came just a few years after the idea [7,8]. Joseph Routin and C. E. Brown patented an electrical-power meter controlled by signals sent over the electrical-power line in 1897 [7].

In 1905, Chester H. Thordarson created an electrical-power meter that sent its values to the network-operation center via an electrical power line. Low-Frequency (below 3 kHz) unidirectional communication systems were installed to low-voltage (LV) and medium-voltage (MV) electrical networks for the power-load control in Germany in 1930 [9]. Such data-transferring systems, used within systems controlling the connection and disconnection of large power loads to an electrical network, became frequently used in the 1950s when Brown Boveri Electric and other companies developed the first PLC system [10]. This PLC system was called Ripple Control System (RCS), and within just a few years after its invention, it was installed in electrical networks in several European countries. These low-frequency high-power signals, produced in the MV network, can go through MV/LV transformers, and reach power consumers connected to the LV network [11]. At the end of 1972, about five million RCS receivers were installed in substations across Europe [12].

The first application of the PLC communication interface for the remote control of power consumption of small consumers was an industry standard named X10 (invented by Pico Electronics in 1975). It enabled the electrical-network operator to control the lighting and other household appliances remotely. X10 was introduced in the US market in 1978, and it can be found in some US distribution networks even today [13,14].

### 1.1. Current Research

Current PLC technologies are classified according to the data-transmission-frequency range that the PLC technologies use into three groups: Ultra-Narrowband PLC (UNB-PLC; operating-frequency range 30 Hz–3 kHz), Narrowband PLC (NB-PLC; operating-frequency range 3–500 kHz), and Broadband PLC (BB-PLC; operating-frequency range 1.8–250 MHz).

Current High Data Rate NB-PLC standards based on Orthogonal Frequency Division Multiplexing (OFDM) operate with the frequency values below 500 kHz (examples of this technology: G3-PLC and Power-line Intelligent Metering Evolution (PRIME)). The theoretical data rates of these communication systems are up to 1 Mbps [15,16], which means that the NB-PLC data rates are sufficient for usage in many SG applications [17].

The quality of the communication is another critical issue. It depends on many factors, such as the noise level (there are background, impulse, synchronous, and asynchronous noises), the signal attenuation (among others, it is caused by impedance mismatches and different cable diameters along the data-transmission path [18]), and the multipath fading (it is caused by the branching of the electrical network between the transmitting and receiving PLC equipment [19]).

Many authors have performed experimental measurements and software simulations of PLC capabilities. Predominantly, these measurements and software simulations were performed for PLC systems installed in LV electrical networks. Compared to MV electrical networks, the LV electrical networks usually have more branches, and here is more frequent appliance switching, which means that there are often impedance changes here, and there are many other signals flowing here that deteriorate the signal produced by the PLC system. When the boom of the PLC-technology research had just started, Hooijen [18] was one of the first researchers who conducted experimental measurement of the PLC-technology capabilities. Specifically, he investigated the PLC technology within the 9 to 95 kHz frequency range installed in the electrical distribution network powering the residential area of Amsterdam, and he explored how the distance between the transmitting PLC unit and the receiving PLC unit affects the data-transmission quality. More recently, Larsson et al. [20] investigated how the increasing number of fluorescent lamps connected to the electrical distribution network affects the 2 to 150 kHz signal spectrum. They identified two different distortions—the recurrent oscillations synchronized with the fundamental-frequency component and the recurrent oscillations synchronized with the high-frequency component. These distortions are generated by the active power-factor correction.

Some authors performed tests comparing more NB-PLC standards. Hoch [21] used MATLAB software to simulate errors in the PLC data transmission where the white Gaussian noise was added to PLC signals. He concluded that the PRIME communication standard is less complex and less robust than G3-PLC.

Mlynek et al. [22] performed the measurement of the data rate of G3-PLC and PRIME communication standards. During the measurements, they explored various modulations of the PLC signals and observed how the various data-transmission distances and streetlamps switching on and off affect the data-transmission quality. They concluded that the PRIME communication standard achieves higher data rates and higher susceptibility to distortion than G3-PLC. Sadowski [23] analyzed the data transmission quality for a PLC system installed in a flat where a personal computer was the only source of distortion. He concluded the same as Mlynek et al.

Other researchers investigated NB-PLC communication channels. Cortés et al. [24] statistically analyzed data-transmission quality values measured during data transmission using PLC via 106 various electrical-power lines (operating in CENELEC A frequency band) to investigate the suitability of its use in an electrical network equipped with the advanced metering infrastructure. The local measurements were performed in three different environments: rural, semi-urban, and urban environment. Within local measurements, the measured values included the magnitude of the noise, the channel response, the input impedance, and the data rate. The measured values were compared to the modelled ones. Cortés et al. concluded that the highest data-transmitting performance was obtained in the semi-urban environment and the lowest in the urban environment. Kharraz et al. [25] measured the impedance variations in the 9–500 kHz frequency band caused by the operation of eight different appliances. They found out the occurrence of short-term and long-term impedance variations.

Furthermore, they pointed out that the impedance of appliances that are not the resistive type of load is frequency dependent. Therefore, the value of the impedance of such appliances may be lower than 1 Ω. The operation of such appliances in an electrical network equipped with the NB-PLC communication system may cause significant problems for a proper data transfer.

Ronnberg et al. [26,27] investigated the interactions between the PLC-system modem and various appliances when the modem and the appliances were connected to the house electrical network. The investigation showed high-frequency (9–95 kHz frequency band) currents flowing only from one device to another, so the currents did not flow to or from the distribution network. They concluded that a capacitor was a source of these high-frequency currents, and this capacitor could be a network-side, installed in an EMC filter, or a DC-side, installed in a diode rectifier. Such results indicate that two appliances operating simultaneously may have a much more significant impact on the PLC system than these two appliances operating separately.

### 1.2. Motivation of Research

The motivation for analyzing the NB-PLC systems in existing electrical networks is to assess if this communication system is suitable for use within an electrical-appliance management system. Errors in the proper data transmission are expected here because the one electrical-power line serves both as the path for the electrical power delivery to the appliances and the data-transmission path for the NB-PLC communication. Therefore, it is needed to explore if an electrical network can operate properly when both the NB-PLC communication system and the electrical appliances are operating here. Although all investigated appliances comply with the EMC standards EN 55011 and EN 55015 (these standards specify the limits of disturbances emitted by domestic electrical appliances, electrical lighting, and similar equipment within the frequency range from 9 kHz to the values way beyond the CENELEC A band), the compliance with the standards does not guarantee that the appliance will not have a negative impact on the proper communication using the PLC system. Especially when it is often necessary to utilize the EMC filter in the electrical network to comply with the limits stated by the standards. As mentioned above, Ronnberg et al. [26,27] stated that EMC filters could be the sources of disturbances for the PLC systems.

Proper management of a small electrical network (i.e., microgrid) requires the use of a reliable communication system in the network. The modern control systems of microgrids control production and consumption of electrical power, electrical appliance switching, electric vehicle (EV) charging/discharging, and connecting the microgrid to the external distribution network (switching between the on-grid and off-grid modes). Within a reliable communication system, the command sent by the control device successfully reaches the controlled device (e.g., switching command sent to the switching device), and the data sent from the end device (e.g., measured data sent by a measuring device) successfully reaches the control device. Unsuccessful data transmission in the microgrid communication system can lead the microgrid to be operated with lower electrical-power quality or outside the defined operational limits, or it can even lead to a failure of some part or the whole microgrid [28,29,30,31].

## 2. Research Objectives

The authors’ previous work in the field of PLC technology describes potential concepts of the real-time communication in off-grid electrical systems [32]. The authors of the paper tested the PLC concepts in their microgrid environment. During the tests, the data transfers were conducted with the use of the PLC system during the operation of one electrical appliance, and the authors of the paper measured the effect of the appliance operation on the data-transfer speed. Within this measurement, the effect of six different appliances was measured.

**Figure 1:**
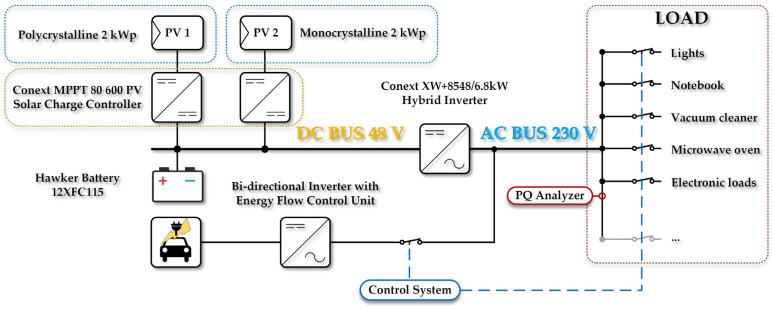
Diagram of the entire test platform [32].

The work presented in this paper follows up on the investigation showed in paper [32] and shifts the PLC research one step forward. This paper presents the results of measuring the influence of the operation of various appliances on the data transmission quality with a PLC system that was created using two PL360 Evaluation Kit modules. Specifically, the value of the Frame Error Ratio (FER) and the Link-Quality Indicator (LQI) were measured here. The local measurements were performed in the experimental microgrid electrical system installed on the campus of the Technical University of Ostrava (see Figure 1). This microgrid is a prototype of a smart electrical network of a house. It can operate both in the on-grid and off-grid modes (this means it may be connected to or disconnected from the external distribution network), and it is equipped with several common household appliances. Using this microgrid system, the authors of this paper have already published several articles that present the measurement results of different aspects connected with the influence of the appliances on, e.g., power quality [33,34,35].

It is worth mentioning that during the usual operation of the presented microgrid electrical system, the local energy consumption and accumulation is controlled using a unique control system. It contains various modules (e.g., the Active Demand Side Management module that controls the local electrical energy consumption to reach an optimal usage of the energy produced by local power sources) [34]. For its proper operation, the local control system needs to communicate with appliances connected to the local microgrid. The authors examined if the local communication system can be realized using a PLC technology-based device. It is worth mentioning that the PLC is open for the implementation of tasks typical for Home Energy Management Systems (HEMS). Both the characteristics of current technical solutions, e.g., data routers that mediate data transmission between individual components or segments and the outside world (IoT), and suitable communication technologies that take advantage of the principle of communication over an electrical network are still being investigated [36,37,38]. In addition, the researchers are engaged in measuring the error rate of transmission and the principles of its reduction on networks with basic household appliances using various commercially available means. This works brings a constructive view of the parameters of NB-PLC communication for the above-mentioned specific power network. The research presented here was also focused on combinations of household appliances where various parameters that affect the hardening of the communication, e.g., the FER error rate parameter, were measured. Some of these combinations, however, have a positive effect on reducing the above-mentioned parameter [39,40].

The main benefits of this study result from the fact that the research was conducted in an environment that was very similar to a real household. It was not a simulation that can lead to new knowledge, for example in the choice of the communication interface for a given system of appliances in ensuring a lower error rate of data by repeating the transmission in a longer time interval. According to the performed real experiments, some combinations led to an improvement of the assessed parameters, other combinations increased the error rate of the transmission, and thus the need for repetition, which prevents or can prevent, in some cases, nominal operating of devices. Then, one of the wireless communication networks, e.g., ZigBee, which may not affect the quality of the data transmission of any home Wi-Fi network, can be recommended. Due to the characteristics of the PLC, the authors believe that it is more secure for operation than wireless networks, which are prone to eavesdropping and more susceptible to possible misuse.

According to the presented overview, the article brings the following innovations:

- Demonstration of the influence of the appliance or combination of appliances on the investigated communication parameters;
- Finding a means to improve the resilience of the PLC communication itself in the off-grid system environment;
- Implementation of communication between the appliances themselves so that they interact with each other regarding the electricity consumption (future work of the authors team).

The paper continues as follows. This section of the paper describes the authors’ microgrid electrical system in which all experiments described here took place. The Section 3 describes the whole local experiment. The Section 4 presents the result of the local analyses. The Section 5 discusses the local observations, and the last section concludes the whole paper.

## 3. Description of the Measurement Platform

**Figure 2:**
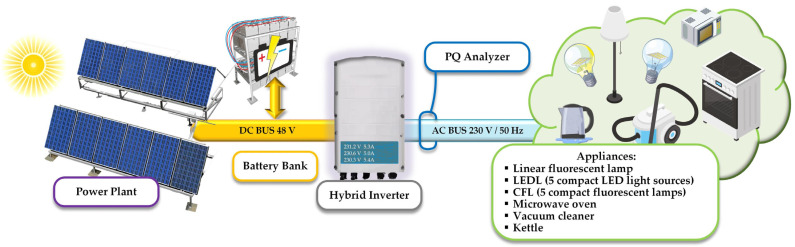
Scheme of the microgrid in which PLC system was tested.

As has already been mentioned in the introductory section, the local measurements were performed in the experimental microgrid electrical system installed on the campus of the Technical University of Ostrava (Figure 2). It was equipped with two photovoltaic arrays (the rated power of each of them is 2 kWp), a hybrid inverter (it is Conext XW+ 8548 with the rated output power of 6.8 kW), a battery electrical-energy storage (it consists of 16 pieces of Hawker 12 XFC battery cell; it is a lead-acid battery cell, its energy capacity is 115 Ah and nominal voltage is 12 V), a system controlling local electrical-power flows, a device measuring the electrical-power quality, and many electrical appliance diagrams of the complete microgrid platform.

To make the local experiments repeatable and to gain a measurement environment here with no phenomenon that could not be described and controlled, within the microgrid during the local experiments, all electrical energy consumed was supplied from a local battery electrical-energy storage and both the photovoltaic arrays and the external distribution network were disconnected from the microgrid. The electrical power was transferred from the energy storage to the hybrid inverter, from the hybrid inverter to the switchboard controlled by the control system, and from the switchboard to local electrical appliances. The control system controls the switching of appliances according to the needs of users with regard to the energy possibilities of the microgrid platform. The system includes appropriate prediction tools for the production of electric energy from PV and an algorithm for managing electricity consumption based on demand side response methods [34]. The current work is focused on developing an extension of the static battery sharing of the microgrid. For this expansion, we want to use the Nissan Leaf electric car with the CHAdeMO protocol. In this solution, the PLC communication for communication of an electric car and the main control system will be used.

### Description of Experiment

**Figure 3:**
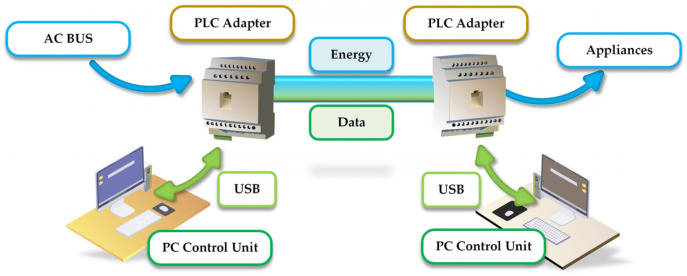
Block diagram of the investigated communication system.

Figure 3 shows the scheme of a PLC communication system used during the local measurements.

Within the measurements, seven groups of appliances were measured, with each of these groups differing in nature. The individual measurements here examined the impact of the operation of each local appliance group on the quality of data transmission when PLC was used. In addition to the measurements when a group of appliances was operated, there was one series of measurements when no appliance was operated. This measurement state is referred to as the no-load state.

Two modules of the PL360 Evaluation Kit were connected to the microgrid to test the PLC communication system. These modules operate with the G3-PLC standard in the CENELEC A frequency band. Table 1 presents the different parameters of this communication system. The values of these parameters were obtained from the user guidance of this communication system.

The individual appliance groups here have been designed to capture the specific nature of all appliances found in the average household. The kettle appliance group represents the typical resistance appliance at home. The vacuum cleaner 1 and 2 appliance groups represent a typical household motorized appliance. In the vacuum cleaner 1 group, there is a vacuum cleaner of an older type than in the vacuum cleaner 2 group. The authors of the article used two vacuum cleaners during the measurements here, as they wanted to find out if the older vacuum cleaner (manufactured before the European Union’s measures to reduce the energy consumption of vacuum cleaners were established) had a different effect on the power grid or different effects on the quality of data transmission via PLC. The microwave oven appliance group represents a resistive load that operates in an intermittent operating mode, i.e., a mode where switch-on and switch-off time periods alternate rapidly. A large part of the power load of a typical home is represented by electric light sources. This is why the other four appliance groups consist only of electric light sources. The group of appliances is made up of five compact LED light sources (LEDL), where there are switching power supplies powering local LEDs. Thus, the LEDL group of appliances represents a typical switched-mode power supply (SMPS) load in a household. The appliance group consists of five compact fluorescent lamps (CFL). In this electrical appliance, the fluorescent lamp is powered by SMPS; however, the SMPS used here is different than the one used in LEDL. In today’s homes, compact fluorescent lamps are being replaced by compact LED lamps; however, there are still many compact fluorescent lamps used. The last two groups of appliances are linear fluorescent lamps 1 and 2. In today’s homes, linear fluorescent lamps are used rarely, but there are rooms where they are frequently found, e.g., garage or cellar. The electric current to linear fluorescent lamps flows through an electrical choke that stabilizes the electric arc. Thanks to the presence of the electrical choke and electrical arc, these electrical appliances have a specific character. Both groups of appliances with linear fluorescent lamps, linear fluorescent lamp 1 and 2, use identical linear fluorescent lamp. The only difference in these two appliance groups is the local electrical choke—each appliance group uses a different electrical choke. Table 2 presents some values describing these electrical appliance combinations.

The operating frequency of linear fluorescent lamp 1 is in the range of 40 to 50 kHz (the authors got these values from the datasheet). The operating frequency of linear fluorescent lamp 2 is in the range of 30.2 to 33.4 kHz (the authors got these values from the measurement of the operation of Linear fluorescent lamp 2). The operating frequency of LEDL is in the range of 23.5 to 25.5 kHz (the authors got these values from the measurement of the LEDL operation). Furthermore, the operating frequency of CFL is in the range of 68 to 81 kHz (the authors got these values from the measurement of the CFL operation).

During experimental measurements, the microgrid was operating in the off-grid mode. The short-circuit power of the microgrid when it is operating in the off-grid mode is lower than the one when it is operating in the on-grid mode. Because of the local low short-circuit performance of the microgrid operating in the off-grid mode, the authors of the paper expected that the impact of the individual groups of appliances on the local data transmission quality would be large.

Each appliance combination was tested three times with different settings of the appliances each time. The first settings of appliances were named normal operation mode, and here, the data was transmitted with no additional measures to counter a possible frame loss. The second set of appliances was named robust operation mode. The third set of appliances was named notch filter operation mode, as the built-in notch filter in the PL360 equipment was activated there. To control the errors in the data transmission, the Forward Error Correction (FEC) method was used here. In the simplest form of FEC, each character was sent twice. The receiver checked both instances of each character for adherence to the protocol being used. If conformity occurred in both instances, the character was accepted. If conformity occurred in one instance and not in another, the character that conformed to the protocol was accepted. If conformity did not occur in either instance, the character was rejected, and a blank space was displayed in its place.

The Forward Error Correction (FEC) comprised the Reed Solomon encoder and the convolutional encoder in normal operation mode. The FEC is composed of the Reed Solomon, the convolutional encoder, and Repetition Code (RC) in the robust operation mode. The RC repeated each bit four times, making the system more robust to channel impairments. However, it reduced the throughput (approximately, by a factor of 4 [41]).

Table 3 presents the individual parameters of these three operating modes. Specifically, Table 3 presents what modulation type, modulation scheme, tone map, transmission interval, local number of frames, frame symbols, local peak, and real effective baud rate were used, and whether the notch filter was applied.

For each tested appliance combination, the test-data transmission was repeated ten times to measure a sufficient amount of data for further analysis. Any appliance here was operating full-time on the test-data transmission. Agilent Signal Analyzer was used to record the average and peak values of the test-data-transmission signal voltage at the transmitter and at the receiver.

## 4. Results of the Research

As was observed, the battery charge level had no significant effect on the results of communication with the use of the PLC devices. The tests were carried out with different charge states of the battery. As part of the experiment, we used batteries in accordance with the manufacturer’s recommendations. The difference in the results was within the tolerance of the measuring devices. There was no trend in this difference.

**Figure 4:**
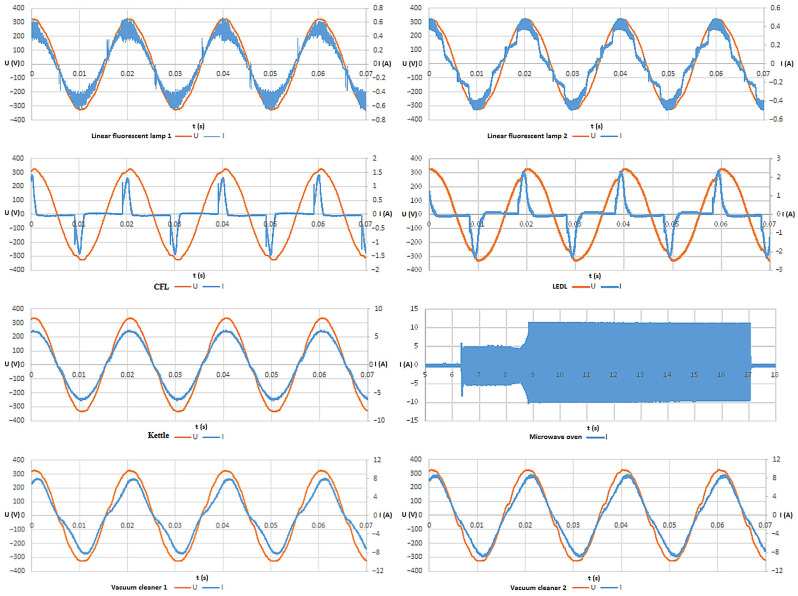
Voltage and current measured during measurement of operation of individual tested appliance combinations (the voltage is presented by the orange lines and the current by the blue lines; the voltage values are presented in volts and the current value in amperes.

**Figure 5:**
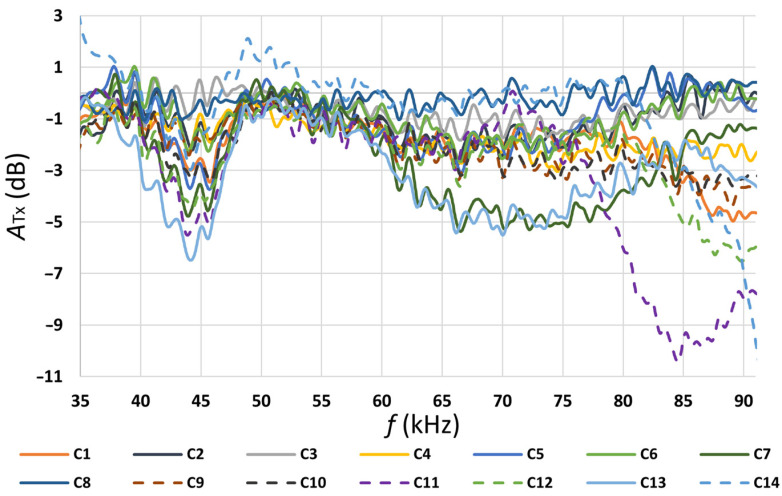
Signal attenuation measured at the transmitter in the function of frequency band.

**Figure 6:**
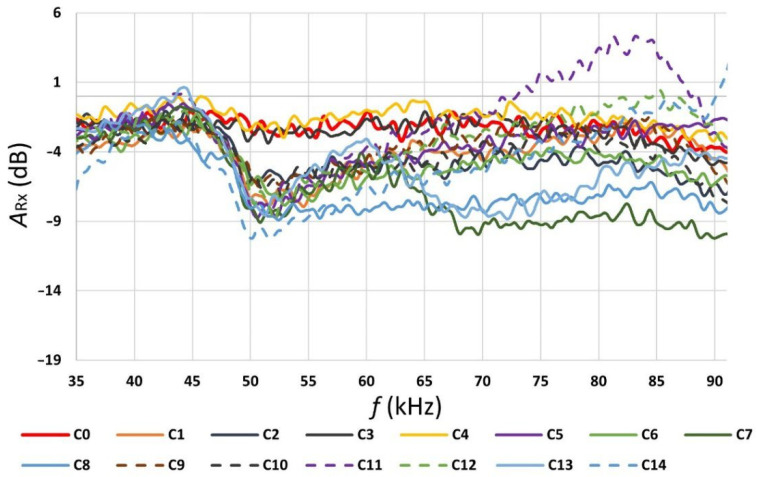
Signal attenuation measured at the receiver in the function of frequency band.

**Figure 7:**
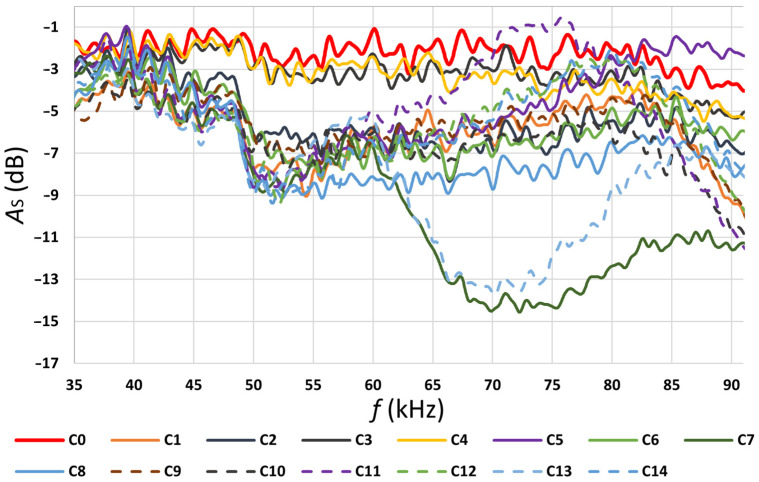
Total signal attenuation (*A*_s_) in the function of frequency band.

The results were measured in PLC settings in a normal mode without the Notch filter. The values measured in the individual operating modes are presented in Figure 4, Figure 5, Figure 6 and Figure 7 and Table 4 and Table 5. In Table 4, the individual appliance groups are classified into groups according to the observed impact of the operation of the appliance group on the data transmission quality. There have been three groups distinguished.

Group 1 contains appliances whose operation has a straight negative effect on the PLC communication system and causes the growth of the FER value. Group 2 contains appliances whose operation has an effect that could be called positive or compensating. Group 3 contains appliances whose operation has a neutral impact on the FER value, having almost no negative effect on FER value. This means that when the appliance is operating alone, it does not cause the FER deterioration. This table shows the values of the following parameters: RS Error—number of frames that Reed Solomon block was able to correct; Bad FCH CRC—number of data frames with invalid CRC frame-control header (the frames with invalid CRC frame-control header are discarded here; Bad payload—number of data frames where the content of payload is not what it is supposed to be.

According to the values shown in Figure 5, the highest attenuation measured at the transmitter was in the frequency range of 45 to 85 kHz of the band plan. According to the values shown in Figure 6, the highest attenuation measured at the receiver was in the frequency range from 50 to 55 kHz. The total attenuation shown in Figure 7 is the sum of attenuation measured at the transmitter and attenuation measured at the receiver. Based on the measured values, a built-in notch filter in the PLC equipment was set at 52 kHz (as seen in Figure 7, the attenuation for frequency 52 kHz was very high).

The data obtained from the measurements contains the frequency characteristics of the signal measured at the signal transmitter output and the signal receiver input and the frequency characteristics of distortion caused by the appliance operation. This data was captured at the time when there was no communication.

According to the impact of the appliance combination operation on the value of the frame error ratio (FER), the appliance combinations were divided into three groups. The group chosen for every appliance combination is presented in Table 4. Appliance combinations when two appliances operated in parallel are classified into two groups, where the individual groups mentioned here correspond to the operation of individual local appliances. The data shown in the last two columns of Table 4 were obtained from the tester tool. FER values were calculated using the following formula:

(1) $$\mathrm{FER}=\frac{n_{sent}-n_{rec}}{n_{sent}}\cdot 100\%$$

In this formula, *n_sent_* is the number of frames transmitted and *n_rec_* is the number of frames correctly received.

The forward link-quality indicator (LQI) is measured for each frame and for the characterization of the underlying power channel quality. LQI value is derived from the average signal-to-noise ratio (SNR) value where averaging is conducted across all active tones and pilot tones presented in the band plan. Active tones are defined as tones that carry data (not including pilot and dummy tones) [16]. LQI takes its values within the range of −10 to 53.75 dB, with the step of 0.25 dB. It is vital to note that the LQI value is not recorded by the tester tool if the distortion is high enough to cause the frame to be lost. Reversely, based on the median and the standard deviation values of LQI, it can be stated that when there is a high distortion (i.e., cases with high FER value), the higher LQI values is necessary to receive the frame.

Table 4 shows the resulting FER values, the median and the standard deviation values of LQI and the LQIIQR/LQISD ratio. The appliances combination with LQISD ≥ 2 and LQIIQR/LQISD ≥ 2 show the multimodal distribution of LQI.

The microwave oven and LEDL are the appliances with this distribution of LQI values. In both appliances combinations, it is explained by the principle of how both appliances operate. See Figure 4.

The operation cycle of such appliances is composed of more than one phase and the time length of at least two phases is comparable. Currents taken from the microgrid during each phase are significantly different. In the appliances’ combinations containing the microwave, it can be easily explained by its way of the microwave operation: there is the electrical motor (that rotates the plate inside) running for 100% of the operation time of the microwave, but there is also the magnetron there, which runs intermittently (the value of the ratio between the length of its running time period and the length of its no-running time period is defined by the current microwave settings related to the heating power). Therefore, the operation period of these combinations should be divided into two phases, and then, these phases should be described separately. During the first phase, there is just the plate-rotating motor running. During the second phase, there is both the plate-rotating motor running and the food heating.

When there are the appliances combinations with LEDL operating, the current taken from the microgrid has a pulse character. Thus, also within the operation of these combinations, there is the phase when a much smaller current is drawn than in the other phases.

Several phases differing in their current drawn can be also seen when the appliances combinations containing CFL were operating. However, the local differences between phases did not affect the LQI value significantly enough to cause the multimodal distribution here.

Within the operation cycle of the linear fluorescent lamp, there are also two phases. During the longer phase (it takes up most of the 20-ms time period), there is an electrical arc in the linear fluorescent lamp, and during the shorter phase, there is no electrical arc in the linear fluorescent lamp. If the operating frequency range of the linear fluorescent lamp overlaps with the communication frequency band of the PLC communication system, then the longer phase causes such a large distortion that the frames of the currently transmitted data are lost.

In the combinations when more than one appliance is operating (C9–C14), it is difficult to observe the multimodal LQI distribution, as there are currents flowing between individual appliances of these appliance combinations [26,27]. These currents do not flow through the microgrid inverter at all, so the affection of the LQI value caused by the operation of local appliances cannot be measured properly.

Table 5 indicates how many data frames were sent and how many data frames were received. The statistics of the received frames that are presented here show how many data frames were received with the Repaired Error (RS Error) and how many data frames were discarded due to unrepairable errors.

Another parameter measured here was *A_Rx_*. It is the ratio (attenuation) of the magnitude of the received signal to the magnitude of the transmitted signal during operation of the investigated appliance, *R_xLoad_* (in Volts) is the magnitude of the signal measured at the receiver, and *T_xLoad_* (in Volts) is the magnitude of the signal measured at the transmitter. *A_Rx_* is defined with the following formula:

(2) $$A_{Rx}=20\cdot \log\frac{R_{xLoad}}{T_{xLoad}}\;\left(\mathrm{dB}\right)$$

In this formula, both parameters, *R_xLoad_* and *T_xLoad_*, were measured during the operation of the investigated appliance combinations.

Another parameter measured here was *A_Tx_*. It is the ratio (attenuation) of the magnitude of the signal transmitted when the investigated appliances combination is operating here, to the magnitude of the signal transmitted when no appliances are connected to the microgrid, *T_xNoLoad_* (in Volts) is the magnitude of the signal measured at the transmitter, while no appliance is connected to the microgrid. *A_Tx_* is defined according to the following formula:

(3) $$A_{Tx}=20\cdot \log\frac{T_{xLoad}}{T_{xNoLoad}}\;\left(\mathrm{dB}\right)$$

Another parameter measured here was *A_s_*. It is the ratio of the magnitude of the received signal *R_xLoad_* to the magnitude of the transmitted signal *T_xNoLoad_*. *A_s_* is defined with the following formula:

(4) $$A_{s}=20\cdot \log\frac{R_{xLoad}}{T_{xNoLoad}}=A_{Tx}+A_{Rx}\left(\mathrm{dB}\right)$$

The values of *A_Rx_*, *A_Tx_*, and *A_S_* are calculated across the whole band plan (range of 35.9 to 90.6 kHz) and show which subcarriers are affected by the distortion generated by running appliances. When all values of *A_S_* had been calculated, they were statistically analyzed.

Another parameter observed here was *Flatness*. This parameter was calculated for *R_x_* and *T_x_*. Flatness of *R_x_* and Flatness of *T_x_* is defined with the following formulas:

(5) $${Flatness}_{Rx}=\frac{\sqrt[{N}]{\Pi_{n=0}^{N-1}Rx\left(n\right)}}{\frac{\Sigma_{n=0}^{N-1}Rx\left(n\right)}{N}},\;\;{Flatness}_{Tx}=\frac{\sqrt[{N}]{\Pi_{n=0}^{N-1}Tx\left(n\right)}}{\frac{\Sigma_{n=0}^{N-1}Tx\left(n\right)}{N}}\;$$

In these two formulas, *R_x_*(*n*), *T_x_*(*n*) are magnitudes of an FFT sample *n* and *N* is the total number of the FFT samples.

The results of the statistical analysis of the total signal attenuation and the *Flatness* values are presented in Table 6. A strong positive correlation between the FER values and the standard deviation values, and a strong negative correlation between the FER values and the minimum can be seen here. The mild negative correlation between the FER values, and *R_x_* and *T_x_* spectral flatness. Such results comply with an assumption that the more deteriorated signal at the receiver, the higher the FER value.

As it is observable from the values provided in the column FER_ROBO_ shows some improvement over FER_Normal_. However, for some appliance combinations, the improvement was not enough to counter the local frame loss. On the other hand, the FER_Notch_ settings improved the reliability of the PLC communication system to the extent that there were no frame losses for any appliance combination (i.e., FER value was 0% for any appliance combination).

Table 7 shows the FER values for all three settings of the PLC equipment, which were used during local measurements.

## 5. Discussion

The investigation of the PLC communication system in the microgrid operating in the off-grid mode showed that appliances could be divided into groups according to the way they affect this communication system.

Group 1 contains appliances whose operation has a straight negative effect on the PLC communication system and causes the growth of the FER value. Linear fluorescent lamp 1, vacuum cleaner 1, and the microwave oven belong to Group 1. Group 3 (electrical kettle, linear fluorescent lamp 2, and vacuum cleaner 2) contains appliances whose operation has a neutral impact on the FER value, having almost no negative effect on FER. Group 2 contains appliances whose operation has an effect that could be called positive or compensating. This means that when the appliance is operating alone, it does not cause the FER deterioration. Their properties (compensation) take effect when operating together with any appliance from Group 1, the outcome is fully or partially negative of the FER growth caused by Group 1 appliances. Signal levels (frequency spectrum) at the transmitter and the receiver side were recorded and used to calculate the spectral flatness and the attenuation across the whole band plan. The total attenuation (*A_s_*) distribution across the whole band plan was statistically described by its median, standard deviation, minimum, maximum, and range.

Then, Spearman’s correlations between the FER values and the spectral flatness (*R_x_* and *T_x_*), the FER values, and the values of the total attenuation (it is the median value and other mentioned statistical values) were calculated. The highest observed value of the correlation was between the FER values and the values of *R_x_* spectral flatness. It was −0.515. This value means a mild to strong negative correlation. The highest observed value of the positive correlation was between the FER values and the standard deviation. It was 0.409. This is a mild positive correlation. Surprisingly, the results showed no correlation between the FER values and the median values. Nevertheless, the median value of the total attenuation should not be considered unimportant for the analysis. It just shows the threshold where its essential value was not reached during the local tests. Such results show that the FER values are more susceptible to uneven distribution of the total attenuation values. The spectral flatness is suitable for describing unevenness.

Possible tools that can be taken to reduce or eliminate the negative impact of the operation of the appliances from Group 1 on the FER values that have been tested. The first tool explored was the utilization of robust BPSK modulation. It reduces the FER values, but not completely, for most appliance combinations. However, this reduction of the FER values also causes the reduction of the bit rate. The second tool was a built-in notch filter in the PLC equipment, which reduced the FER values in all combinations to 0% with no measurable bit-rate reduction. Another possible tool is to insert an additional EMC filter to the point of connecting the problematic appliance to the microgrid. This tool increases the installation cost, and it is not universally applicable within any appliance group, for example, within the group with linear fluorescent lamps equipped with electrical choke.

## 6. Conclusions

The previously presented discussion on the achieved research results revealed that some household appliances used in the electrical network may significantly affect the data transfer through that network. Furthermore, the use of special filters here can considerably improve the quality of communication in this electrical line. On this broadly stated basis, specific conclusions can be drawn in the form of the following statements:

- Without a connected appliance, the FER value was 0. The communication was therefore error-free. It can be stated that the interference of the hybrid converter without load does not affect the communication;
- The applied linear fluorescent lamp 1 has a major effect on the PLC communication, the FER value reached the level of 75.39%. While using linear fluorescent lamp 2 caused the FER value of 0. It can be stated that the selection of low-quality linear fluorescent lamps can cause potential communication problems;
- An older vacuum cleaner 1 has better FER values than a newer vacuum cleaner 2. This is due to the different electronic design of the vacuum cleaner;
- The microwave affects PLC communication. This is due to the cyclic switching of the inductive load;
- The combination of LEDL and linear fluorescent lamp 1 has a positive effect on PLC communication. From the experiment, it can be stated that some combinations of appliances have a positive effect on the durability of the PLC;
- Combinations of some appliances, on the other hand, have a negative effect on PLC communication. In the experiment, these combinations of linear fluorescent lamp 1 + vacuum cleaner 1, linear fluorescent lamp 1 + microwave oven were tested, and the worst combination appeared to be that of linear fluorescent lamp 1 + kettle;
- ROBO modulation has a major impact on improving the FER parameter and thus on the PLC communication. The difference is especially noticeable for the appliance combinations where the FER value is high;
- PLC communication with the Notch filter leads to an FER value of 0 for all appliance combinations. The correct setting of the notch filter leads to a robust communication solution with narrowband power-Line communication in the off-grid electrical system.

On the basis of the presented conclusions, it may be assumed that future works will be focused on investigating the operation of a greater number of appliances of the same type, for example, LEDL, because there is a great number of them available on the market. Therefore, there would be a higher possibility for the creation of an appliance group whose operating frequency would be inside the frequency band of the PLC system communication, and thus, the operation of this appliance group would possibly cause the FER-value deterioration. The next phenomenon that should be investigated is the impact of PV operation on the communication quality in the microgrid. It would be also interesting to investigate the effect of appliance operation on the FER values while the PLC communication system is communicating in CENELEC B or CENELEC C frequency bands (these frequency bands are dedicated to general use and for the domestic needs, respectively).

## Figures and Tables

**Table 1:** Parameters of PL360 Evaluation kit—G3-PLC.

Parameters	Type, Value
Modulation schemes	Coherent or differential
Modulation types	Robust BPSK, BPSK, QPSK, or 8PSK
1st subcarrier	35.9 kHz
Last subcarrier	90.6 kHz
Subcarrier spacing	1.562 kHz
Number of subcarriers	36
Sub-bands	6
Error encoding decoding	Viterbi, Reed Solomon, convolution, or CRC16

**Table 2:** Various parameters of the measured appliance combinations.

Appliance	Load (VA)			Power Factor (-)			Appliance Type	EMC Filter
	Avg	Min	Max	Avg	Min	Max		
Linear fluorescent lamp 1	67.1	63.4	70.3	0.992	0.991	0.993	Load with electrical choke	Yes
Linear fluorescent lamp 2	65.5	64.9	65.9	0.969	0.967	0.970	Load with electrical choke	Yes
LEDL (5 pieces)	78.8	79.8	80.6	0.494	0.487	0.501	Switched-mode-power-supply load	Yes
CFL (5 pieces)	81.1	80.5	81.8	0.546	0.538	0.551	Electronic-ballast load	Yes
Microwave oven	692.7	42.7	1312.3	0.806	0.232	0.95	Switched resistive load	Yes
Vacuum cleaner 1 (2015)	993.0	973.5	1032.4	0.985	0.981	0.985	Load with universal electrical motor	Yes
Vacuum cleaner 2 (2008)	1183.3	1123.5	1201.5	0.991	0.985	0.992	Load with universal electrical motor	Yes
Kettle	1053	1048	1062	0.995	0.994	0.996	Resistive load	No

**Table 3:** Transmission session settings.

Parameters	1st (Normal)	2nd (Robust)	3rd (Notch Filter)
Modulation type	BPSK	ROBO	BPSK
Modulation scheme	Differential	Differential	Differential
Tone map	0x3F	0x3F	0x3F
Transmission interval	100 ms	100 ms	100 ms
Number of frames	1000	1000	1000
Number of frame characters	65	65	65
Effective baud rate (peak)	15,296 kbps	5181 kbps	15,296 kbps
Effective baud rate (real)	7810 kbps	4690 kbps	7810 kbps
Notch filter	No	No	Yes

**Table 4:** Combination used to test PLC and results from tester tool (Med = median, SD = standard deviation, IQR = interquartile range).

Combination	Appliance	Group (Combo)	FER (%)	LQI Med (dB)	LQI SD (dB)	LQI IQR/LQI SD
C0	No appliance (no-load state)	-	0.00	10.75	3.78	0.595
C1	Linear fluorescent lamp 1	1	75.39	8.75	1.15	0.870
C2	Linear fluorescent lamp 2	3	0.00	6.75	0.89	1.124
C3	LEDL	2	0.00	4.00	5.64	1.95
C4	CFL	2	0.00	5.25	3.40	1.103
C5	Vacuum cleaner 1	1	73.00	11.25	1.67	1.048
C6	Vacuum cleaner 2	3	0.25	14.5	0.54	1.389
C7	Microwave oven	1	8.88	9.25	2.23	2.018
C8	Kettle	3	0.71	12.5	0.63	1.587
C9	Linear fluorescent lamp 1 + LEDL	1 + 2	7.54	5.00	2.35	0.957
C10	Linear fluorescent lamp 1 + CFL	1 + 2	0.00	6.75	1.20	1.042
C11	Linear fluorescent lamp 1 + vacuum cleaner 1	1 + 1	87.28	13.75	1.57	1.433
C12	Linear fluorescent lamp 1 + vacuum cleaner 2	1 + 3	0.00	14.51	0.74	0.676
C13	Linear fluorescent lamp 1 + microwave oven	1 + 1	84.06	9.75	2.21	1.131
C14	Linear fluorescent lamp 1 + kettle	1 + 3	88.52	13.5	1.78	1.404

**Table 5:** Numbers (*N*) of transmitted and received (OK, corrected, and discarded) frames and calculated Frame Error Ratio (FER).

Combination	*N* of Frames Transmitted	*N* of Received Frames—OK	*N* of Corrected Frames	*N* of Received-Discarded Frames (dB)		FER (%)
				Bad FCH CRC	Bad Payload	
C0	13,977	13,977	0	0	0	0.00
C1	19,356	4764	3	955	6	75.39
C2	9994	9994	0	0	0	0.00
C3	9987	9987	241	0	0	0.00
C4	9985	9985	0	0	0	0.00
C5	9992	2700	1	317	2	72.98
C6	9938	9913	2	0	0	0.25
C7	11,993	10,927	0	65	1	8.89
C8	9981	9910	0	0	0	0.71
C9	21,850	20,240	118	52	1	7.37
C10	12,962	12,962	8	0	0	0.00
C11	7657	973	2	465	5	87.29
C12	9984	9984	0	0	0	0.00
C13	8985	1434	1	1535	16	84.04
C14	9779	1123	2	376	6	88.52

**Table 6:** Statistical analysis of *A_s_* in dB and spearman’s correlation between FER and signal describing parameters (statistical and flatness).

**Combination**	**Median**	**SD**	**Minimum**	**Maximum**	**Range**	**Spectral Flatness**		**Group**
						***R_x_*** **(dB)**	***T_x_*** **(dB)**	
C0	−2.13	0.63	−3.90	−1.07	2.82	−0.0068	−0.0076	
C1	−5.64	1.40	−9.45	−3.12	6.33	−0.0197	−0.0081	1
C2	−5.89	1.49	−8.34	−1.05	7.29	−0.0191	−0.0109	3
C3	−3.11	0.81	−5.33	−1.59	3.74	−0.007	−0.0077	2
C4	−3.15	0.96	−5.49	−1.21	4.28	−0.0073	−0.0069	2
C5	−4.66	2.09	−8.73	−0.95	7.78	−0.0367	−0.0155	1
C6	−9.96	3.66	−14.57	−2.37	12.20	−0.046	−0.0143	3
C7	−6.09	1.47	−8.22	−2.08	6.15	−0.0212	−0.0107	1
C8	−7.56	1.96	−9.14	−1.45	7.69	−0.0291	−0.0091	3
C9	−5.44	1.22	−9.31	−2.91	6.40	−0.0154	−0.006	1 + 2
C10	−6.24	1.37	−10.66	−3.45	7.21	−0.0146	−0.006	1 + 2
C11	−4.94	2.51	−11.14	−0.54	10.60	−0.0327	−0.0184	1 + 1
C12	−9.28	4.07	−15.92	−1.86	14.06	−0.0272	−0.0092	1 + 3
C13	−5.36	1.76	−9.47	−2.47	7.01	−0.0325	−0.0113	1 + 1
C14	−5.27	1.97	−9.38	−1.95	7.43	−0.0068	−0.0076	1 + 3
Spearman’s correlation	−0.024	0.409	−0.314	−0.089	0.251	−0.515	−0.452	

**Table 7:** Frame error ratio for all three PLC settings.

Combination	Appliance	FER_Normal_ (%)	FER_ROBO_ (%)	FER_Notch_ (%)
C0	No appliance (no-load state)	0.00	0.00	0.00
C1	Linear fluorescent lamp 1	75.39	17.53	0.00
C2	Linear fluorescent lamp 2	0.00	0.00	0.00
C3	LEDL	0.00	0.00	0.00
C4	CFL	0.00	0.00	0.00
C5	Vacuum cleaner 1	72.98	13.15	0.00
C6	Vacuum cleaner 2	0.25	0.00	0.00
C7	Microwave oven	8.89	0.25	0.00
C8	Kettle	0.71	0.00	0.00
C9	Linear fluorescent lamp 1 + LEDL	7.37	0.81	0.00
C10	Linear fluorescent lamp 1 + CFL	0.00	0.00	0.00
C11	Linear fluorescent lamp 1 + vacuum cleaner 1	87.29	7.94	0.00
C12	Linear fluorescent lamp 1 + vacuum cleaner 2	0.00	0.00	0.00
C13	Linear fluorescent lamp 1 + microwave oven	84.04	42.03	0.00
C14	Linear fluorescent lamp 1 + kettle	88.52	5.19	0.00

## Data Availability

Not applicable.

## References

[1] Wu N.E., Sarailoo M., Salman M. (2018). Transmission Fault Diagnosis with Sensor-Localized Filter Models for Complexity Reduction. IEEE Trans. Smart Grid.

[2] Lisowski M., Masnicki R., Mindykowski J. (2019). PLC-Enabled Low Voltage Distribution Network Topology Monitoring. IEEE Trans. Smart Grid.

[3] Chen C., Chen Y., Ding N., Wang Y., Lin J.-C., Zeng X., Huang D. (2013). Accurate Sampling Timing Acquisition for Baseband OFDM Power-Line Communication in Non-Gaussian Noise. IEEE Trans. Commun..

[4] Shlezinger N., Dabora R. (2014). Frequency-Shift Filtering for OFDM Signal Recovery in Narrowband Power Line Communications. IEEE Trans. Commun..

[5] Shlezinger N., Dabora R. (2015). On the Capacity of Narrowband PLC Channels. IEEE Trans. Commun..

[6] Ahola J. (2003). Applicability of Power-Line Communications to Data Transfer of Online Condition Monitoring of Electrical Drives. Ph.D. Thesis.

[7] Windelspecht M. (2003). Groundbreaking Scientific Experiments, Inventions, and Discoveries of the 19th Century.

[8] Brown P.A. Power Line Communications-Past, Present, and Future. Proceedings of the International Symposium on Power-line Communications and its Applications (ISPLC ‘99).

[9] Doster K.M. Telecommunications over the Power Distribution Grid—Possibilities and Limitations. Proceedings of the IEEE International Symposium on Power Line Communications and Its Applications, (ISPLC ‘97).

[10] Dzung D., von Hoff T., Stoupis J., Kranich M. (2010). Connected, the Nervous System of the Smart Grid.

[11] Dzung D., Berganza I., Sendin A. (2011). Evolution of Powerline Communications for Smart Distribution: From Ripple Control to OFDM. Proceedings of the 2011 IEEE International Symposium on Power Line Communications and Its Applications.

[12] Morgan M.G., Talukdar S.N. (1979). Electric Power Load Management: Some Technical, Economic, Regulatory and Social Issues. Proc. IEEE.

[13] Rye Dave X10 Technology, Hometoys Inc. 1 October 1999. https://hometoys.com/dave-rye-x10/.

[14] Fritz R. (2019). Is X-10 an Obsolete Technology? There Are Better and Newer Technologies to Use. Lifewire.

[15] Telecommunication Standardization Sector of ITU (2014). Narrowband Orthogonal Frequency Division Multiplexing Power Line Communication Transceivers for G3-PLC Networks.

[16] Berganza I., Bois S., Brunschweiler A., PRIME Alliance Technical Working Group (2014). PRIMEv1.4 White Paper. https://www.prime-alliance.org/wp-content/uploads/2020/04/whitePaperPrimeV1p4_final.pdf.

[17] Casella I.R., Anpalagan A. (2018). Power Line Communication Systems for Smart Grids.

[18] Hooijen O.G. (1998). On the Channel Capacity of the Residential Power Circuit Used as a Digital Communications Medium. IEEE Commun. Lett..

[19] Berger L.T., Schwager A., Escudero-Garzás J.J. (2013). Power Line Communications for Smart Grid Applications. J. Electr. Comput. Eng..

[20] Larsson E.O.A., Bollen M.H.J. (2010). Measurement Result from 1 to 48 Fluorescent Lamps in the Frequency Range 2 to 150 KHz. Proceedings of the 14th International Conference on Harmonics and Quality of Power—ICHQP 2010.

[21] Hoch M. (2011). Comparison of PLC G3 and PRIME. Proceedings of the 2011 IEEE International Symposium on Power Line Communications and Its Applications.

[22] Mlynek P., Koutny M., Misurec J., Kolka Z. (2014). Measurements and Evaluation of PLC Modem with G3 and PRIME Standards for Street Lighting Control. Proceedings of the 18th IEEE International Symposium on Power Line Communications and Its Applications.

[23] Sadowski Z. (2015). Comparison of PLC-PRIME and PLC-G3 Protocols. Proceedings of the 2015 International School on Nonsinusoidal Currents and Compensation (ISNCC).

[24] Cortés J.A., Sanz A., Estopiñán P., García J.I. (2015). Analysis of Narrowband Power Line Communication Channels for Advanced Metering Infrastructure. EURASIP J. Adv. Signal Process..

[25] Kharraz M.A.O., Lavenu C., Jensen P., Picard D., Serhir M. (2017). Characterization of the Input Impedance of Household Appliances in the FCC Frequency Band. Proceedings of the 2017 IEEE International Symposium on Power Line Communications and its Applications (ISPLC).

[26] Ronnberg S.K., Bollen M.H.J., Wahlberg M. (2011). Interaction Between Narrowband Power-Line Communication and End-User Equipment. IEEE Trans. Power Deliv..

[27] Ronnberg S.K., Wahlberg M., Larsson E.O.A., Bollen M.H.J., Lundmark C.M. (2009). Interaction between Equipment and Power Line Communication: 9–95 KHz. Proceedings of the 2009 IEEE Bucharest PowerTech.

[28] Zezulka F., Fiedler P., Bradáč Z., Šír M. (2010). Trends in Automation—Investigation in Network Control Systems and Sensor Networks.

[29] Zezulka F., Bradáč Z., Sajdl O., Šembera J. (2012). Experimental Smart Grid. IFAC Proc. Vol..

[30] Kaczmarczyk V., Bradac Z., Fiedler P. (2017). A Heuristic Algorithm to Compute Multimodal Criterial Function Weights for Demand Management in Residential Areas. Energies.

[31] Da Rocha Farias L., Monteiro L., Leme M., Stevan S. (2018). Empirical Analysis of the Communication in Industrial Environment Based on G3-Power Line Communication and Influences from Electrical Grid. Electronics.

[32] Blazek V., Petruzela M., Vysocky J., Prokop L., Misak S., Seidl D. (2020). Concept of Real-Time Communication in Off-Grid System with Vehicle-to-Home Technology. Proceedings of the 2020 21st International Scientific Conference on Electric Power Engineering (EPE).

[33] Blazek V., Petruzela M., Vantuch T., Slanina Z., Mišák S., Walendziuk W. (2020). The Estimation of the Influence of Household Appliances on the Power Quality in a Microgrid System. Energies.

[34] Mišák S., Stuchlý J., Platoš J., Krömer P. (2015). A Heuristic Approach to Active Demand Side Management in Off-Grid Systems Operated in a Smart-Grid Environment. Energy Build..

[35] Vantuch T., Misak S., Jezowicz T., Burianek T., Snasel V. (2017). The Power Quality Forecasting Model for Off-Grid System Supported by Multiobjective Optimization. IEEE Trans. Ind. Electron..

[36] Yu D., Li K., Yu S., Trinh H., Zhang P., Oo A.M.T., Hu Y. (2021). A Novel Power and Signal Composite Modulation Approach to Powerline Data Communication for SRM in Distributed Power Grids. IEEE Trans. Power Electron..

[37] Ikpehai A., Adebisi B., Rabie K., Haggar R., Baker M. (2016). Experimental Study of 6LoPLC for Home Energy Management Systems. Energies.

[38] Ali K., Liu A.X., Pefkianakis I., Kim K.-H. (2018). Distributed Spectrum Sharing for Enterprise Powerline Communication Networks. Proceedings of the 2018 IEEE 26th International Conference on Network Protocols (ICNP).

[39] Wasowski M., Sikorski T., Wisniewski G., Kostyla P., Szymanda J., Habrych M., Gornicki L., Sokol J., Jurczyk M. (2021). The Impact of Supply Voltage Waveform Distortion on Non-Intentional Emission in the Frequency Range 2–150 KHz: An Experimental Study with Power-Line Communication and Selected End-User Equipment. Energies.

[40] Sausen P.S., Sausen A., de Campos M., Sauthier L.F., Oliveira A.C., Emmel Junior R.R. (2021). Power Line Communication Applied in a Typical Brazilian Urban Power Network. IEEE Access.

[41] Electricite Reseau Distribution France (2009). G3-PLC Physical Layer Specificatio.

